# Insulin and IGF-1 have both overlapping and distinct effects on CD4^+^ T cell mitochondria, metabolism, and function

**DOI:** 10.1038/s41598-024-54836-w

**Published:** 2024-02-21

**Authors:** Kaitlin Kiernan, Yazan Alwarawrah, Amanda G. Nichols, Keiko Danzaki, Nancie J. MacIver

**Affiliations:** 1 Department of Immunology, Duke University School of Medicine, Durham, NC USA; 2 Department of Pediatrics, Division of Pediatric Endocrinology, University of North Carolina School of Medicine, Chapel Hill, NC USA; 3 Department of Nutrition, School of Medicine and Gillings School of Global Public Health, University of North Carolina, Chapel Hill, NC USA

**Keywords:** Lymphocytes, Biochemistry, Hormones

## Abstract

Insulin and insulin-like growth factor 1 (IGF-1) are metabolic hormones with known effects on CD4^+^ T cells through insulin receptor (IR) and IGF-1 receptor (IGF-1R) signaling. Here, we describe specific and distinct roles for these hormones and receptors. We have found that IGF-1R, but not IR, expression is increased following CD4^+^ T cell activation or following differentiation toward Th17 cells. Although both insulin and IGF-1 increase the metabolism of CD4^+^ T cells, insulin has a more potent effect. However, IGF-1 has a unique role and acts specifically on Th17 cells to increase IL-17 production and Th17 cell metabolism. Furthermore, IGF-1 decreases mitochondrial membrane potential and mitochondrial reactive oxygen species (mROS) in Th17 cells, providing a cytoprotective effect. Interestingly, both IR and IGF-1R are required for this effect of IGF-1 on mitochondria, which suggests that the hybrid IR/IGF-1R may be required for mediating the effect of IGF-1 on mitochondrial membrane potential and mROS production.

## Introduction

Insulin and insulin-like growth factor 1 (IGF-1) are closely related hormones that regulate different aspects of growth and metabolism. Insulin is secreted primarily from pancreatic beta cells in proportion to blood glucose levels and is best known for promoting glucose uptake in insulin-responsive metabolic cells and tissues^1^. In individuals with obesity, serum insulin levels may be increased due to peripheral insulin resistance^1^. Although structurally similar to insulin, IGF-1 is produced primarily in the liver in response to growth hormone signaling and is best known for promoting growth^2^. However, IGF-1 can also be secreted from other cells and tissues and regulated by protein levels, such that IGF-1 is low in states of protein malnutrition^3,4^. Conversely, free IGF-1 is elevated in obesity due to decreased levels of IGF binding proteins^5–7^. In addition to its effects on growth, IGF-1 can also regulate apoptosis and cellular metabolism and has been implicated in the regulation of aging and inflammation^8–13^.

Insulin and IGF-1 signal through insulin receptor (IR) and IGF-1 receptor (IGF-1R), respectively. Like the hormones, IR and IGF-1R are also structurally similar, allowing both insulin and IGF-1 to bind and signal through each other’s receptors, albeit at lower efficiency^14–16^. Furthermore, IR and IGF-1R chains can heterodimerize to form a hybrid receptor through which both hormones can signal, although IGF-1 has higher affinity^17,18^. While IR and IGF-1R share many downstream signaling pathways, there are some preferential differences between phospho-signaling downstream of the two receptors that contribute to unique effects of both the hormones and hormone receptors^16,19^.

In previous studies, both insulin and IGF-1 signaling have been shown to affect CD4^+^ T cell metabolism and function. First, CD4^+^ T cells from Wistar rats with whole body knockdown of insulin receptor were found to have reduced cytokine production, reduced proliferation, and increased apoptosis^20^. Second, in mouse studies, insulin was found to promote CD4^+^ T cell production of IFN-γ, as well as both glycolytic and oxidative metabolism^21^. In this study, T cell specific loss of IR signaling also caused reduced antigen-specific influenza responses^21^. Third, IGF-1 was found to increase both cellular metabolism and IL-17 production in Th17 cells, as measured by extracellular flux analysis and flow cytometry, respectively^22^. Specifically, IGF-1 treatment of Th17 cells increased glycolytic and oxidative metabolism, whereas T cell specific IGF-1R knockout was protective against disease in the experimental autoimmune encephalomyelitis (EAE) model^22^. In non-T cells, IGF-1 has also been described as cytoprotective, given its ability to preserve mitochondrial function and biogenesis^23–25^, but this has yet to be studied in T cells.

Here, we identify both overlapping and distinct effects of insulin and IGF-1 on CD4^+^ T cell mitochondria, metabolism, and function. We report the novel finding that IR levels are not dynamically regulated by CD4^+^ T cell activation, but IGF-1R levels are. We confirm that both insulin and IGF-1 modulate CD4^+^ T cell metabolism, although insulin is generally more potent in that regard, but only IGF-1 promotes Th17 cell metabolism and IL-17 production. However, we also report the novel finding that both IR and IGF-1R are required to mediate IGF-1 effects on Th17 cell function, suggesting that IGF-1 is signaling through the hybrid IR/IGF-1R, which has not been described on CD4^+^ T cells to date. Moreover, we show that IGF-1 treatment of CD4^+^ and Th17 cells decreases mitochondrial membrane potential and mitochondrial reactive oxygen species (mROS) production in both an IGF-1R and IR dependent manner, again suggesting that the hybrid IR/IGF-1R may be required for mediating the effect of IGF-1 on mitochondrial membrane potential and mROS production. Overall, these results suggest a cytoprotective role for IGF-1 in CD4^+^ T cells, particularly Th17 cells.

## Results

### CD4^+^ T cell activation leads to increased IGF-1R, but not IR, expression

**Figure 1:**
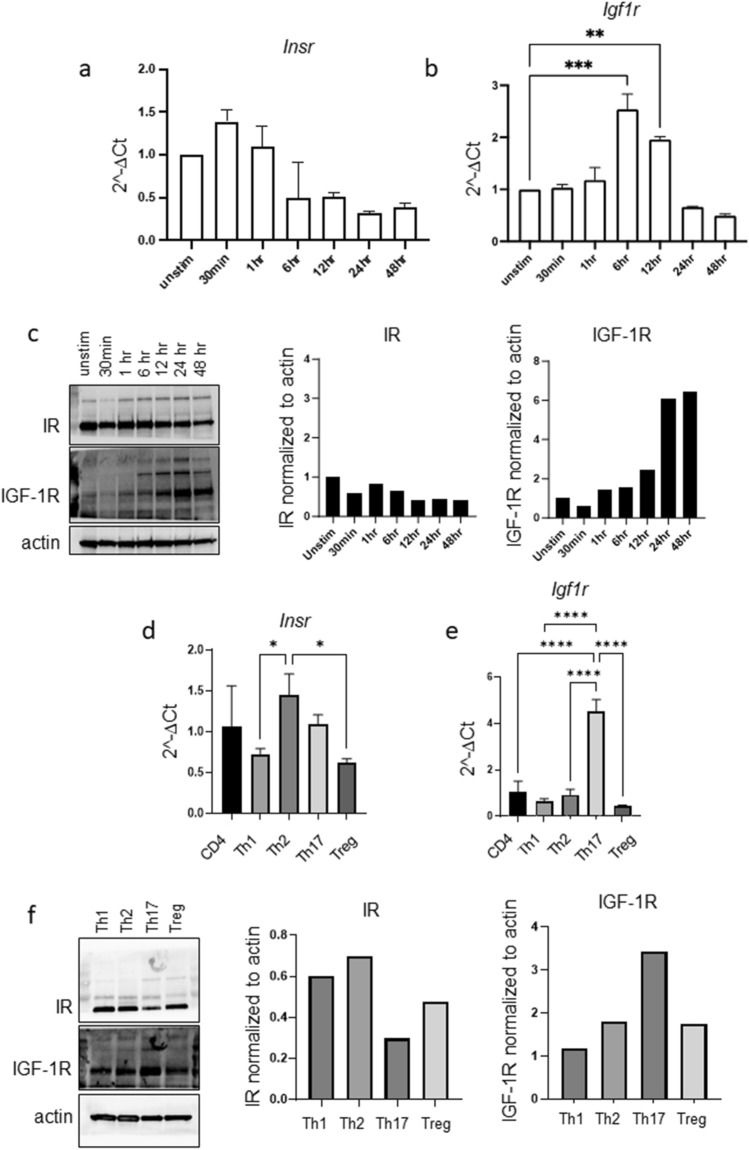
IGF-1R, but not IR, is dynamically regulated by CD4^+^ T cell activation and differentiation. (**a**–**c**) CD4^+^ T cells were activated with anti-CD3 and anti-CD28 antibodies for 0 h (unstimulated), 30 min, 1 h, 6 h, 12 h, 24 h, 48 h. Expression of *Insr (a)* and *Igf1r (b)* mRNA was analyzed by RT-qPCR. Separate samples were analyzed for IR and IGF-1R protein expression by immunoblot *(c)*. (**d**–**f**) CD4^**+**^ T cells were differentiated in vitro to functional T helper subsets. Expression of *Insr (d)* and *Igf1r (e)* mRNA was analyzed by RT-qPCR. Separate samples were analyzed for IR and IGF-1R protein expression by immunoblot *(f)*. Results representative of three independent experiments. Data analyzed by one way ANOVA. In Fig. 1a-b, all experimental points are compared only to the unstimulated condition. (*p < 0.05; **p < 0.01, ***p < 0.001 ****p < 0.0001).

Previous publications demonstrating that CD4^+^ T cells have increased binding of fluorescently labeled insulin following activation have led to the assumption that T cell activation increases IR expression^21,26,27^. However, insulin can bind both IR and IGF-1R, albeit with a lower affinity for IGF-1R^28^. Thus, we examined both *Insr* and *Igf1r* gene expression following CD4^+^ T cell activation. CD4^+^ T cells were isolated from splenocytes of wildtype C57BL/6J mice and activated for up to 48 h with anti-CD3 and anti-CD28 antibodies. Interestingly, we observed that *Insr* gene expression was not significantly changed over the course of activation; however, *Igf1r* gene expression was dynamically upregulated, with mRNA expression peaking at 6 h post activation (Fig. 1a,b). To support these results, we also examined protein expression of IR and IGF-1R following CD4^+^ T cell activation using immunoblot and found that IR protein levels were unchanged following CD4^+^ T cell activation, whereas IGF-1R protein levels were indeed upregulated following activation and accumulated over time (Fig. 1c).

We next interrogated the gene expression levels of *Insr* and *Igf1r* on CD4^+^ T helper cell subsets (Th1, Th2, Th17, Treg), which we generated in vitro by activating splenic CD4^+^ T cells in the presence of cytokines and antibodies that promote subset differentiation, as previously described^29^. Each functional subset showed unique gene expression of *Insr* and *Igf1r* (Fig. 1d,e). Notably, there was a striking increase in *Igf1r* expression in Th17 cells compared to the other CD4^+^ T helper subsets. These results were consistent with protein expression, as determined by immunoblot, which confirmed that Th17 cells have the highest expression of IGF-1R protein among the CD4^+^ T helper subsets (Fig. 1f).

### IGF-1 treatment increases IL-17 production by CD4^+^ T cells

**Figure 2:**
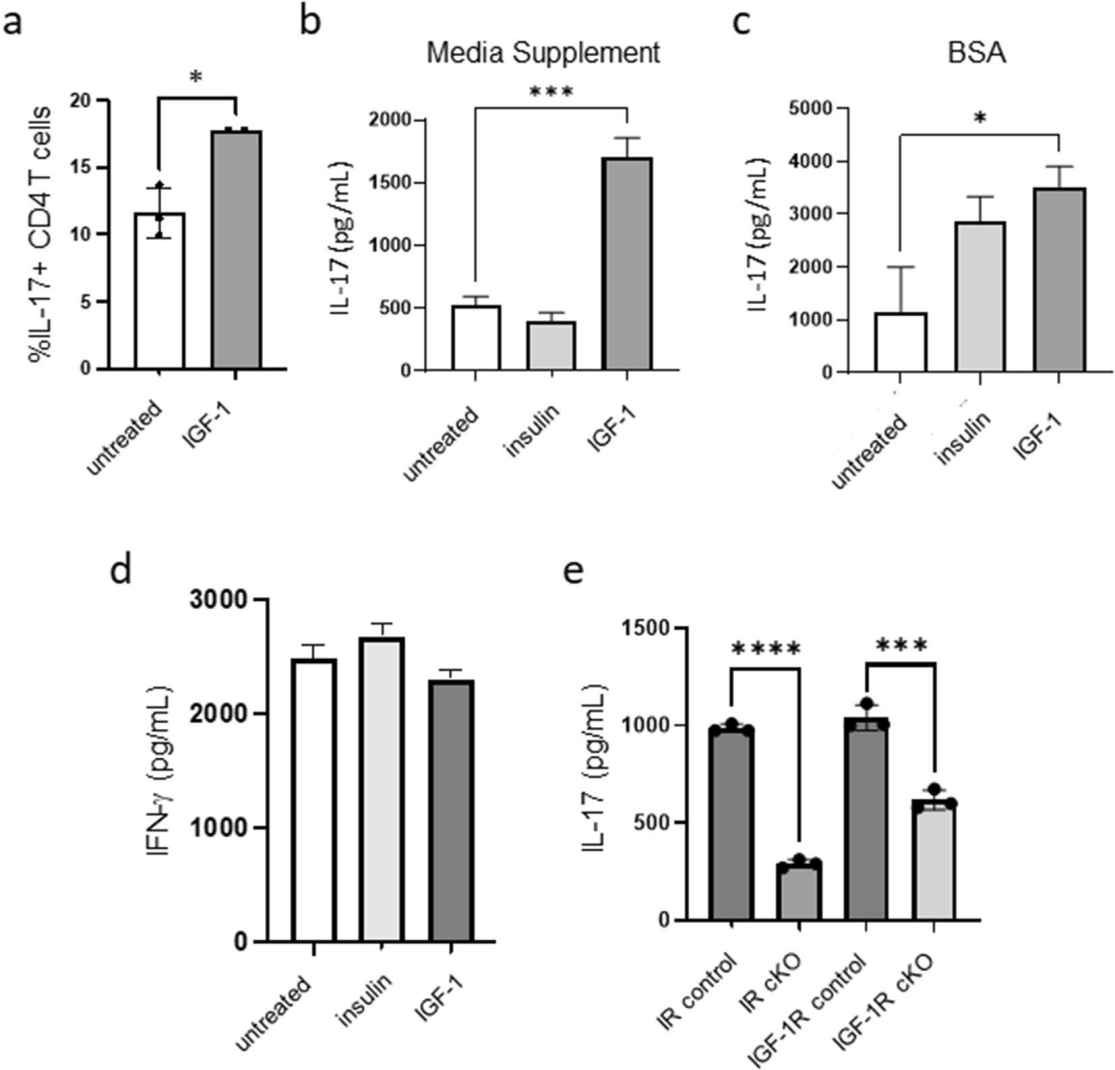
IGF-1 treatment increases IL-17 production by CD4^+^ T cells. (**a**) CD4^+^ T cells were activated for 48 h on anti-CD3/CD28 coated plates in serum free conditions in the presence or absence of IGF-1 for the last 24 h of activation, after which IL-17 expression was analyzed by flow cytometry. (**b**, **c**) CD4^**+**^ T cells were activated for 48 h on anti-CD3/CD28 coated plates in media supplemented with either Insulin Free Media Supplement *(b)* or 0.35% BSA *(c)* in the presence or absence of insulin or IGF-1 for the last 24 h, after which IL-17 production was measured by ELISA. (**d**) CD4^**+**^ T cells were activated for 48 h on anti-CD3/CD28 coated plates in media supplemented with Insulin Free Media Supplement in the presence or absence of insulin or IGF-1 for the last 24 h, after which IFN-γ production was measured by ELISA. (**e**) Splenic CD4^**+**^ T cells from IR cKO or IGF-1R cKO and littermate control mice were activated for 48 h in full serum conditions, after which IL-17 production was measured by ELISA. Data representative of at least 2 independent experiments; n = 3 mice per experiment. Data analyzed using student’s t-test (*p < 0.05; ***p < 0.001 ****p < 0.0001).

Given the increased expression of IGF-1R on Th17 cells, we next asked if IGF-1 would increase T cell production of IL-17 in vitro. To test this, splenic CD4^+^ T cells were isolated from wildtype C57BL/6J mice and activated with plate-bound antibodies to CD3 and CD28 in serum free media supplemented with either Insulin Free Media Supplement or bovine serum albumin (BSA) to prevent any confounding effect of growth factors or hormones found in serum. Following 24 h of activation, cells were treated with or without 50 ng/mL IGF-1, and IL-17 production was examined at 48 h. We found that IGF-1 increased the percentage of CD4^+^ T cells producing IL-17, as measured by flow cytometry (Fig. 2a), as well as the production of IL-17 by CD4^+^ T cells, as measured by ELISA (Fig. 2b,c), whereas insulin treatment did not significantly increase the production of IL-17 (Fig. 2b,c). Furthermore, neither insulin nor IGF-1 altered IFN-γ production by activated CD4^+^ T cells (Fig. 2d). We also observed no changes in viability, cell size, activation marker expression, or proliferation in CD4^+^ T cells activated in the presence of insulin or IGF-1 (Supplementary Fig. S1).

To understand whether this effect of IGF-1 on IL-17 production is dependent on IR or IGF-1R signaling, we activated splenic CD4^+^ T cells from IR conditional knockout (cKO) mice (*CD4Cre*^+^*IR*^*fl/fl*^) or IGF-1R cKO mice (*CD4Cre*^+^*IGF1R*^*fl/fl*^) and littermate controls in full serum conditions and measured IL-17 production by ELISA. IGF-1 was present in the serum during the activation, but no additional IGF-1 was added. Activated CD4^+^ T cells from both IR cKO and IGF-1R cKO mice showed reduced IL-17 production, suggesting that both IR and IGF-1R signaling are required for activated CD4^+^ T cells to produce IL-17 (Fig. 2e).

### Both insulin and IGF-1 modulate CD4^+^ T cell metabolism

**Figure 3:**
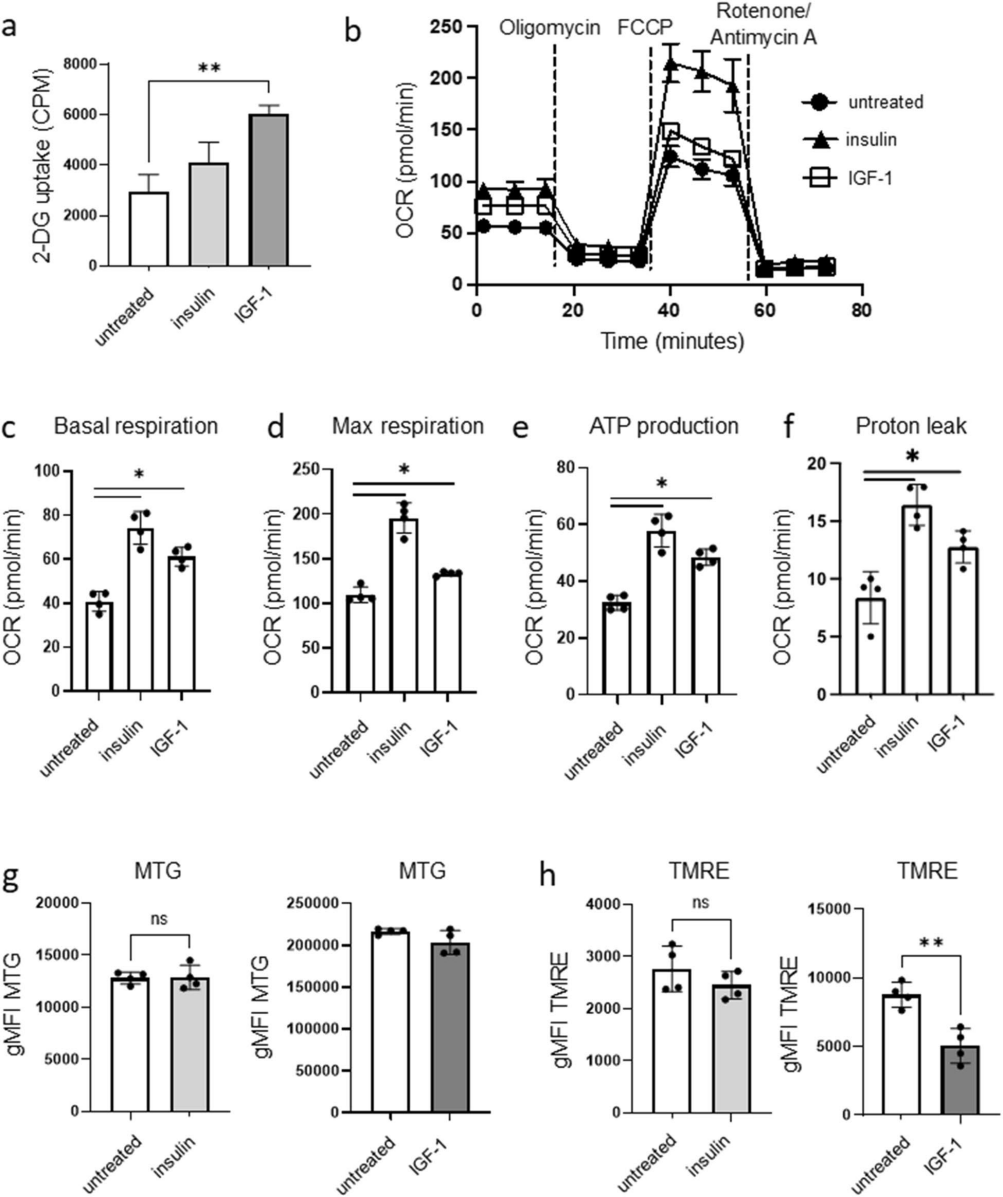
Both insulin and IGF-1 modulate CD4^+^ T cell metabolism. CD4^**+**^ T cells from C57BL/6 mice were activated on anti-CD3/CD28 coated plates for 48 h in media supplemented with Insulin Free Media Supplement, in the presence or absence of insulin (10 ng/mL) or IGF-1 (50 ng/mL) for the last 24 h of activation. (**a**) Glucose uptake was measured by uptake of tritiated 2-deoxyglucose. (**b**–**f**) extracellular flux analysis and Seahorse Mito Stress test were used to measure basal OCR, max OCR, ATP production, and protein leak. (**g**) Mitochondrial mass was measured by MitoTracker Green geometric mean fluorescence intensity (gMFI) using flow cytometry. (**h**) Mitochondrial membrane potential was measured by TMRE gMFI using flow cytometry. Data representative of at least 2 independent experiments; n = 3–4 mice per experiment. Data analyzed using student’s t-test (*p < 0.05; **p < 0.01).

Both insulin and IGF-1 alter the metabolism of metabolic cells and tissues, with insulin particularly well known for increasing glucose uptake^1,9,13^; thus, we next examined and compared the effects of insulin and IGF-1 on CD4^+^ T cell metabolism. Treatment of activated splenic CD4^+^ T cells with a physiological dose of IGF-1 increased glucose uptake, as measured by relative uptake of tritiated 2-deoxyglucose normalized to cell number, while treatment with a physiological dose of insulin did not (Fig. 3a). However, insulin significantly increased basal oxygen consumption rate (OCR; a surrogate for mitochondrial metabolism), maximal respiration, ATP production, and proton leak in activated CD4^+^ T cells, as measured using extracellular flux analysis and using the Seahorse Mito Stress test (Fig. 3b–f). Similar to insulin, IGF-1 treatment of activated CD4^+^ T cells also significantly increased basal respiration, maximal respiration, ATP production, and proton leak (Fig. 3b–f). Overall, however, the metabolic changes in activated CD4^+^ T cells treated with 50 ng/mL IGF-1 were less potent than the metabolic changes seen in activated CD4^+^ T cells treated with 10 ng/mL insulin.

### IGF-1 uniquely impacts the mitochondrial membrane potential of activated CD4^+^ T cells

IGF-1 has been implicated in several mitochondrial processes such as mitochondrial biogenesis, mitophagy, and mitochondrial function^23–25^. We therefore measured the mitochondrial mass and mitochondrial membrane potential of activated CD4^+^ T cells treated with insulin or IGF-1. First, activated CD4^+^ T cells were stained with MitoTracker Green (MTG) to measure mitochondrial mass. Neither insulin nor IGF-1 caused any significant change in mitochondrial mass compared to untreated cells (Fig. 3g). In order to generate ATP in the mitochondria, mitochondrial membrane potential is generated by pumping protons into the intermembrane space which creates a gradient and polarizes the inner mitochondrial membrane. This proton gradient is then used to generate ATP via ATP synthase. To measure the degree of inner mitochondrial membrane polarization, we stained cells with tetramethylrhodamine (TMRE), which is a cell permeant positively charged dye that accumulates in active mitochondria due to their relative negative charge. Inactive or depolarized mitochondria with decreased membrane potential fail to sequester TMRE. IGF-1 treatment of activated CD4^+^ T cells significantly decreased TMRE staining, while insulin treatment had no significant effect (Fig. 3h), suggesting that IGF-1, but not insulin, decreases mitochondrial membrane potential in activated CD4^+^ T cells.

### IGF-1 treatment of Th17 cells promotes metabolism and function

**Figure 4:**
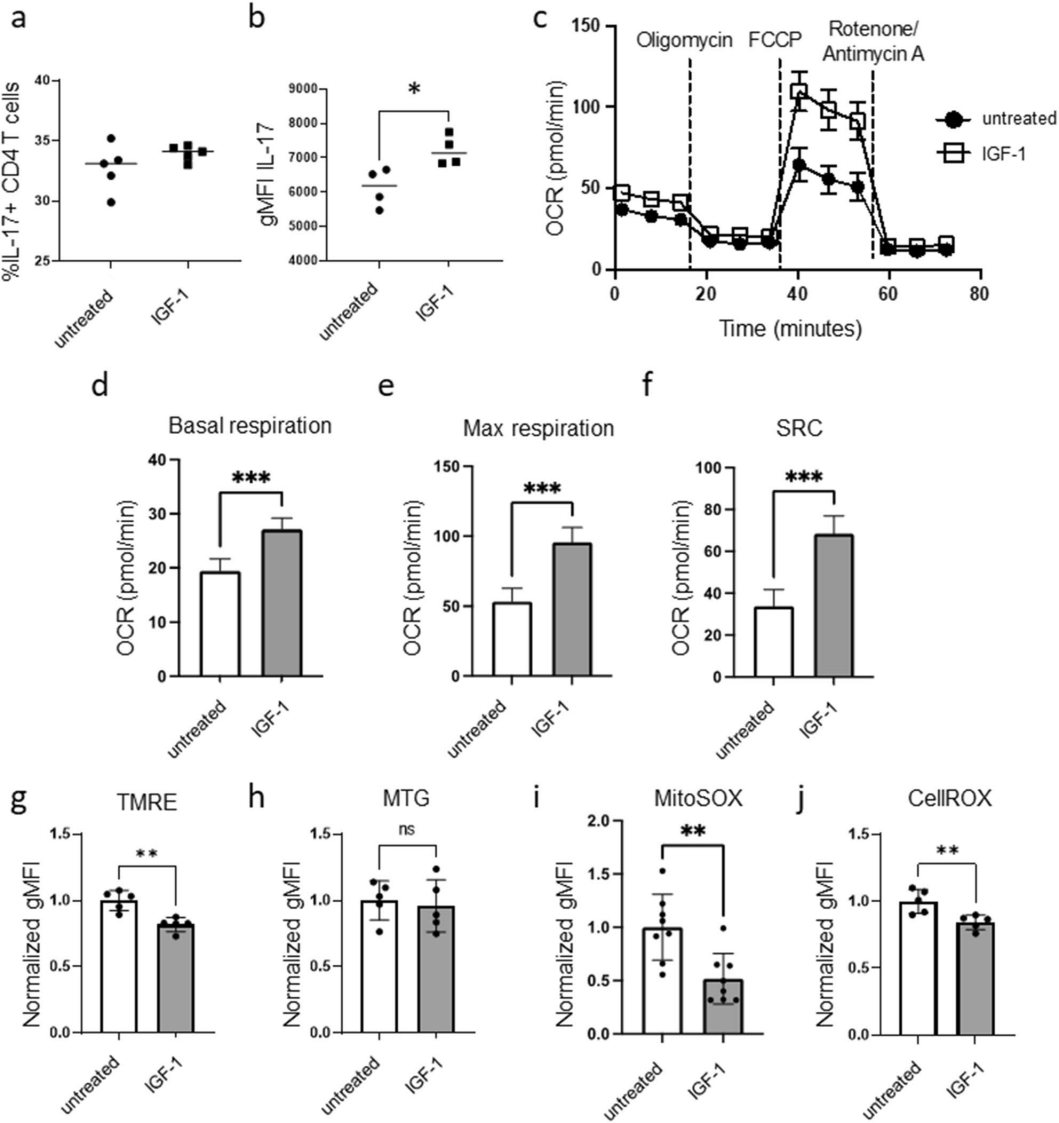
IGF-1 influences Th17 mitochondria, metabolism, and function. CD4^+^ T cells from C57BL/6 mice were differentiated in vitro to Th17 cells in media supplemented with Insulin Free Media Supplement for 3 days, and then treated with or without IGF-1 (50 ng/mL) for an additional 48 h. (**a**, **b**) Cells were analyzed by flow cytometry for percent IL-17^+^ cells *(a)* and IL-17 expression by MFI *(b)*. (**c**–**f**) Extracellular flux analysis and Seahorse Mito Stress test were used to measure basal OCR, max OCR, and SRC. (**g**) Mitochondrial membrane potential measured by TMRE staining, normalized to untreated gMFI. (**h**) Mitochondrial mass measured by mitotracker green staining, normalized to untreated gMFI. (**i**) mROS measured by MitoSOX staining, normalized to untreated gMFI. (**j**) Cellular ROS production measured by CellROX staining, normalized to untreated gMFI. (**a**–**f**) Data representative of 3 independent experiments. n = 4–5 mice per experiment. (**g**–**j**) Data combined from 2–3 independent experiments. n = 5–7 mice/group. Data analyzed using student’s t test (*p < 0.05; **p < 0.01, ***p < 0.001).

Given our findings that IGF-1R is significantly upregulated on Th17 cells compared to other CD4^+^ T helper subsets (Fig. 1e–f), and treatment with IGF-1 causes an increase in IL-17 production by activated bulk CD4^+^ T cells (Fig. 2a–c), we next investigated the effect of IGF-1 treatment on the function and metabolism of Th17 cells. Th17 cells were differentiated from CD4^+^ T cells in vitro, as previously described^29^, and then treated with or without IGF-1 for an additional 48 h. IGF-1 treatment of differentiated Th17 cells did not increase the percentage of IL-17 positive cells but did increase IL-17 production as measured by mean fluorescent intensity flow cytometrically (Fig. 4a,b). IGF-1 also altered the metabolism of differentiated Th17 cells, with increased basal OCR, maximal OCR, spare respiratory capacity (SRC), ATP production, and proton leak (Fig. 4c–f and Supplementary Fig. S2), mirroring the results seen in bulk activated CD4^+^ T cells treated with IGF-1. In contrast, differentiated Th1 and Treg cells treated with IGF-1 did not show any change in IFN-γ or Foxp3 expression, respectively, nor increased cellular metabolism (Supplementary Fig. S3).

### IGF-1 treatment of Th17 cells reduces mitochondrial membrane potential and mROS

IGF-1 treated Th17 cells also had decreased mitochondrial membrane potential compared to untreated Th17 cells, as measured by TMRE staining (Fig. 4g), but showed no significant difference in mitochondrial mass (Fig. 4h). Decreased membrane polarization can be an indicator that the mitochondria are uncoupled, meaning that protons are translocating across the inner mitochondrial membrane through a mechanism other than ATP synthase, and are thus not used to make ATP. This mechanism of uncoupling can be used by cells to protect against oxidative stress since increased mitochondrial polarization causes an increase in the production of ROS. To test whether IGF-1 treatment of Th17 cells decreased mROS along with decreased mitochondrial membrane potential, we measured mROS using the MitoSOX stain. Indeed, we saw that Th17 cells treated with IGF-1 had reduced MitoSOX staining (Fig. 4i). Furthermore, cellular ROS production, as measured by CellROX staining, also showed decreased ROS production in IGF-1 treated Th17 cells (Fig. 4j), suggesting that IGF-1 exerts a cytoprotective effect in addition to a metabolic effect.

### IGF-1 treatment of Th17 cells decreases mitochondrial membrane potential and mROS production in an IGF-1R and IR dependent manner

**Figure 5:**
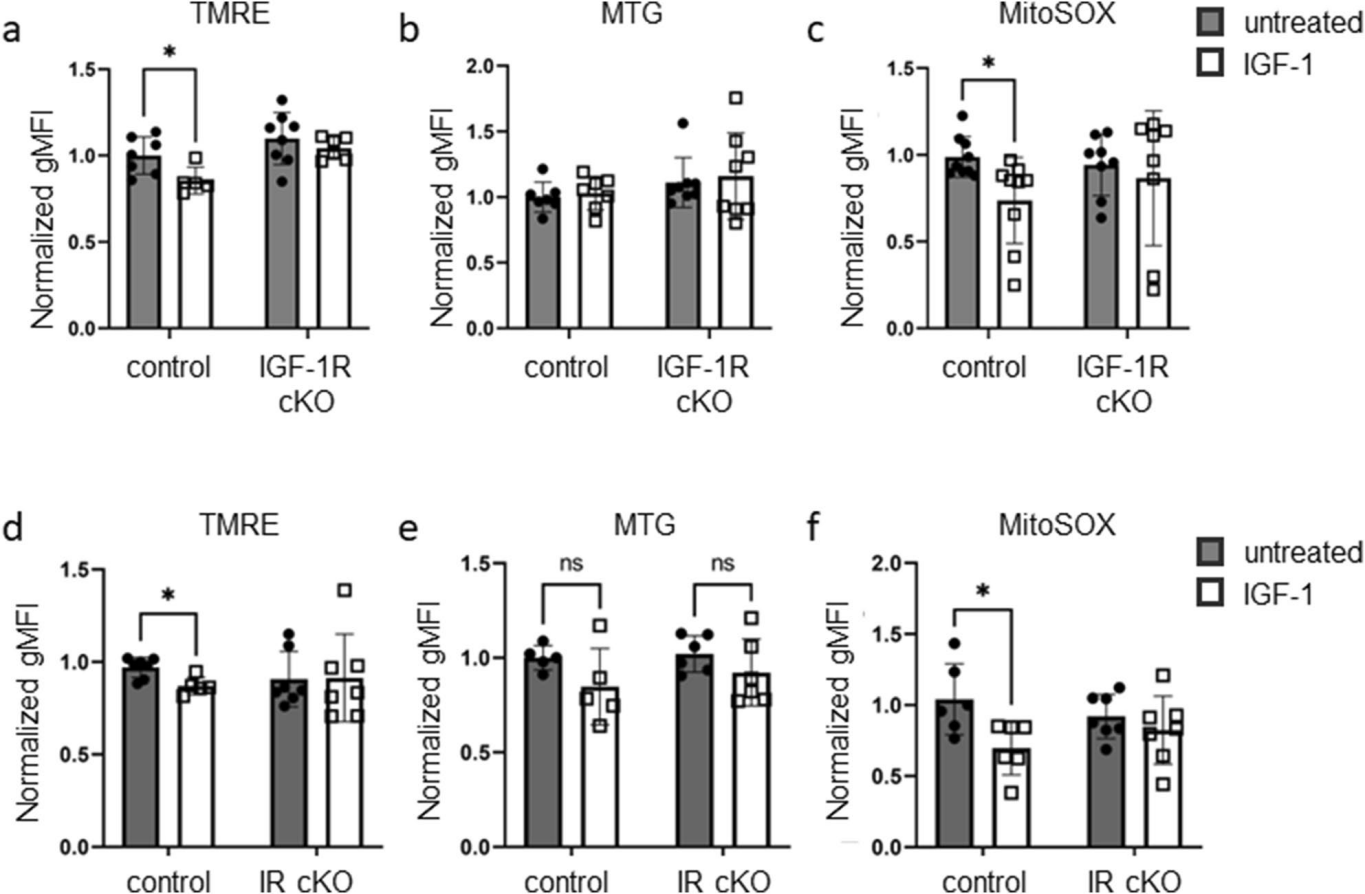
IGF-1 treatment of Th17 cells decreases mitochondrial membrane potential and mROS production in an IGF-1R and IR dependent manner. (**a**–**c**) CD4^+^ T cells were isolated from IGF-1R cKO mice and littermate controls and differentiated in vitro to Th17 cells in media supplemented with Insulin Free Media Supplement, then treated with or without IGF-1 (50 ng/mL) for an additional 48 h. Mitochondrial membrane potential measured by TMRE staining; normalized geometric mean fluorescence intensity shown *(a)*. Mitochondrial mass measured by MTG staining; normalized geometric mean fluorescence intensity shown *(b)*. mROS measured by MitoSOX staining; normalized geometric mean fluorescence intensity shown *(c)*. (**d**–**f**) CD4^**+**^ T cells were isolated from IR cKO mice and littermate controls and differentiated in vitro to Th17 cells in media supplemented with Insulin Free Media Supplement, then treated with or without IGF-1 (50 ng/mL) for an additional 48 h. Mitochondrial membrane potential measured by TMRE staining; normalized geometric mean fluorescence intensity shown *(d)*. Mitochondrial mass measured by MTG staining; normalized geometric mean fluorescence intensity shown *(e)*. mROS measured by MitoSOX staining; normalized geometric mean fluorescence intensity shown *(f)*. Data combined from three independent experiments. (**a**–**c**) n = 5–8 mice per group (**d**–**f**) n = 5–7 mice per group. Data analyzed using student’s t-test (*p < 0.05).

To determine if these IGF-1-mediated changes on Th17 cell mitochondrial membrane potential and mROS require signaling by IR, IGF-1R, or both, we differentiated Th17 cells in vitro from IGF-1R cKO mice or IR cKO mice, and littermate controls. Following differentiation, Th17 cells were treated with or without IGF-1 for an additional 48 h. We observed that IGF-1 treatment of Th17 cells from IGF-1R cKO mice did not decrease TMRE, as it did in Th17 cells from control mice (Fig. 5a). Consistent with our earlier results, mitochondrial mass was not significantly changed in either control or IGF-1R cKO Th17 cells treated with IGF-1 (Fig. 5b). Similar to TMRE, MitoSOX staining was decreased in control Th17 cells but not in Th17 cells generated from IGF-1R cKO mice (Fig. 5c). These data suggest that the effect of IGF-1 on Th17 cell mitochondrial membrane polarization and mROS production is IGF-1R dependent.

Since IGF-1R and IR are highly homologous and are both able to bind IGF-1, we also investigated the requirement for IR in mediating the effect of IGF-1 on mitochondrial membrane potential and mROS production in Th17 cells. IGF-1 treatment of Th17 cells from IR cKO mice did not decrease mitochondrial membrane potential as it did in Th17 cells from control mice (Fig. 5d). Mitochondrial mass was not significantly changed following IGF-1 treatment of Th17 cells from either control or IR cKO mice (Fig. 5e). Lastly, IGF-1 treatment of Th17 cells from IR cKO mice did not decrease mROS as it did in Th17 cells from control mice (Fig. 5f). Interestingly, these results suggest that the effect of IGF-1 on Th17 cell mitochondrial membrane polarization and mROS production require both IR and IGF-1R, and therefore indicate a potential role for the IR/IGF-1R hybrid in mediating effects of IGF-1 on Th17 cell mitochondria.

## Discussion

Previous studies have independently shown the effects of insulin and IGF-1 on CD4^+^ T cell metabolism and function^20–22^. Given the high degree of homology of IR and IGF-1R, their overlapping signaling pathways, and the potential for insulin and IGF-1 to signal through both receptors, as well as the hybrid receptor^14–19^, we set out to understand the relationship between these hormones and receptors on CD4^+^ T cells. Here we present data suggesting that IGF-1 receptor expression is upregulated upon CD4^+^ T cell activation at both the protein and transcript level (Fig. 1). This contrasts with previous studies using ligand binding assays that reported increased insulin receptor expression upon activation^21,26,27^. Since insulin can bind to IGF-1 receptor, it is likely that at saturated staining conditions, changes in IGF-1 receptor expression were detected using this method.

We also show side-by-side the metabolic and functional effects of insulin and IGF-1 on activated CD4^+^ T cells (Figs. 2, 3). Our data suggest that insulin has a more potent metabolic effect on CD4^+^ T cells at physiologic concentrations (Fig. 3a–h). In contrast, IGF-1 has a more specific effect on Th17 cells and IL-17 production (Figs. 2a–c, 4). Furthermore, our data demonstrate that the metabolic reprogramming downstream of insulin or IGF-1 signaling is maintained following 24–48 h exposure, which differs from previous studies that examined the response to these hormones using serum starvation followed by acute hormone treatment^21,22^. This suggests that nutritional status communicated to T cells via changes in insulin and IGF-1 hormone levels results in a metabolic and functional program that is persistent and maintained. This has clinical relevance, as both insulin and free IGF-1 levels are increased in individuals with obesity, and T cells have been found to dominate peripheral inflammation in obesity in human studies, with Th17 cells having a particularly prominent role^30–32^.

While investigating whether IGF-1 impacted mitochondrial biogenesis and function in CD4^+^ T cells, we found that the mitochondrial membrane potential (TMRE staining) was reliably decreased downstream of IGF-1 treatment. Polarization of the mitochondria is very tightly controlled, and depolarization can indicate proton leakage back across the mitochondrial membrane via decoupling of the electron transport chain from ATP production by ATP synthase^33^. Reduced mitochondrial membrane potential could indicate non-functional mitochondria^34^, but our extracellular flux analyses demonstrate that the mitochondria are performing oxidative phosphorylation at an increased rate in IGF-1 treated cells. We therefore investigated another potential reason for decreased mitochondrial membrane potential, which is protection against mROS generation^35^. Using the MitoSOX probe, we found that IGF-1 treatment reduced mROS levels in Th17 cells. Despite this probe being widely used for the measurement of mROS^36,37^, we acknowledge that there are limitations to its use, including the potential to detect changes in non-mitochondrial specific ROS^37,38^; however, we believe these results are still informative as to the production of ROS within cells in response to IGF-1.

Interestingly, both IR and IGF-1R appear to be required for the effects of IGF-1 on Th17 cell mitochondrial metabolism and cytokine production (Figs. 2e, 5), which could indicate that the hybrid receptor is required for mediating the effects of IGF-1 on Th17 cells. The hybrid IR/IGF-1R has been shown to form in various cell lines^17^; however, the presence of hybrid receptor on CD4^+^ T cells has not been established. The hybrid IR/IGF-1R also has a higher affinity for IGF-1 than insulin^18^ but may have actions independent of either IR signaling or IGF-1R signaling alone^19^. Further studies using double knockout mice and/or co-immunoprecipitation/proximity ligation assay are required to definitively establish the presence of hybrid receptor and the requirement for both IR and IGF-1R in this process.

Our model is as follows: IGF-1, signaling through IR and/or IGF-1R, signals to the T cell to increase cellular metabolism. In Th17 cells in particular, IGF-1 signaling also reduces mitochondrial membrane potential and reduce ROS production, which is particularly damaging to Th17 cells. This cytoprotective effect of IGF-1 could be critical for Th17 cells, specifically, as Th17 cells have been shown to be sensitive to ROS signaling^39^. Thus, IGF-1 signaling on Th17 cells may have evolved to protect the Th17 response from potential damage due to high metabolic flux.

## Methods

### Animals

All mouse studies were performed on the C57BL/6 background. T cell-specific insulin receptor (IR) and IGF-1 receptor (IGF-1R) conditional knockout (cKO) mice were generated by crossing CD4Cre transgenic mice with IR-floxed and IGF-1R-floxed mice (Jackson Laboratory, Bar Harbor, ME). Mice were backcrossed for ten generations to the C57BL/6 background. The following genotypes were studied: *CD4Cre*^+^*IR*^*fl/fl*^ (IR cKO) and *CD4Cre*^*-*^*IR*^*fl/fl*^ (controls), *CD4Cre*^+^*IGF-1R*^*fl/fl*^ (IGF-1R cKO) and *CD4Cre*^*-*^*IGF-1R*^*fl/fl*^ (controls). Mice were group housed (up to 5 per cage), maintained at ambient temperature, and given ad libitum access to food and water. Experimental mice were used at 8–12 weeks of age. All animal experiments were approved by the Institutional Animal Care and Use Committees at Duke University or the University of North Carolina at Chapel Hill and our reporting follows the recommendations in the ARRIVE guidelines. All methods were carried out in accordance with relevant guidelines and regulations.

### Tissue collection, processing, and cell culture

Mice were euthanized using CO_2_ inhalation. Spleens were mashed and strained in PBS, washed, and resuspended. CD4^+^ T cells were isolated from splenocytes using the StemCell CD4^+^ T cell isolation kit (STEMCELL Technologies, Vancouver, BC, Canada). Isolated CD4^+^ T cells were activated for 48 h on a plate that was coated overnight with anti-CD3 (1 µg/mL; BioLegend, San Diego, CA) and anti-CD28 (5 µg/mL; BioLegend) antibodies. Where indicated, CD4^+^ T cells were treated with insulin (10 ng/mL) or IGF-1 (50 ng/mL) for the last 24 h of activation. Activated CD4^+^ T cells were analyzed by flow cytometry or extracellular flux analysis. Serum free conditions were used to minimize the impact of hormones and growth factors in media influencing results. RPMI 1640 with glutamine and sodium bicarbonate (2 g/L Glucose) (Sigma-Aldrich, cat#R8758) was supplemented with additional glutamine (Thermo, cat#25030149) (final concentration 40 mM), 10 mM HEPES (Thermo, cat#15630106), 1X penicillin–streptomycin (Thermo, cat#15140122), 0.1% 2-mercaptoethanol (Thermo, cat#21985023) and either 0.35% BSA (Sigma-Aldrich, cat# A4161), or N21-MAX Insulin Free Media Supplement (R&D systems, cat# AR010) (1×), which is certified insulin and glucose free. CD4^+^ T cells were plated at 1 million cells per mL. To skew CD4^+^ T cells to functional subsets of T helper cells, the protocol described by Espinosa et al.^29^ was followed.

### RT-qPCR

RNA was isolated from cell pellets using RNeasy kits (Qiagen, Germantown, MD) following manufacturer’s instructions. cDNA was made from isolated RNA using Bio-Rad iScript cDNA synthesis kits (Bio-Rad, Hercules, CA). To quantify *Insr* and *Igf1r* expression, the following primers were used: *Insr* forward: AATGGCAACATCACACACTACC; *Insr* reverse: CAGCCCTTTGAGACAATAATCC; *Igf1r* forward: AGAACGCCGACCTCTGTTACCTC; *Igf1r* reverse: GCTTGTTCCCCACAATGTAGTT. The reactions were carried out in 384 well PCR plates in a total volume of 4 µl containing 10 µM of reverse and forward primers, 2 µl of SensiFAST Sybr lo ROX RT-PCR mix (Bioline, Boston, MA), and 5 ng of template cDNA (1 µl). qPCR was run on a Bio-Rad CFX Maestro.

### Western blot

Cell pellets were lysed with RIPA buffer plus protease and phosphatase inhibitors for 20–40 min on ice, vortexing every 5 min. The pellets were then spun down for 30 min at high speed and the supernatants collected. Protein concentration was determined by colorimetric protein assay. Protein was diluted to 1 µg/µL in lysis buffer and 5× sample buffer. 35 µL of protein was loaded onto an 8–15% gel, with 8 µL ladder on either side of samples. Gel was run for 40 min at 200 V. Gel was transferred onto a PVDF membrane (Bio-Rad) using the Bio-Rad transfer case. Membrane was blocked overnight in 5% BSA in TBST on a shaker at 4 °C. Membrane was then blotted with antibodies to either IR (Cell Signaling Technology, Danvers, MA, Cat#3025), IGF-1R (Cell Signaling Technology, Cat#9750) or β-actin (Cell Signaling Technology, Cat#4970) for 1 h at room temperature, up to overnight at 4 °C. Blots were imaged on a Bio-Rad gel imager and analyzed using Bio-Rad ImageLab software.

### Flow cytometry

For viability staining, CD4^+^ T cells were washed with PBS then stained with Zombie viability dyes (BioLegend) at room temperature for 5 min, and then washed with FACS buffer. For intracellular cytokine staining, at least 1 million splenocytes were stimulated for 4.5 h in complete media containing Golgi Plug (2 μg/ml) (BD Biosciences, San Jose, CA), PMA (50 ng/ml) (Sigma-Aldrich, St. Louis, MO), and ionomycin (1 μg/ml) (Sigma-Aldrich), then permeabilized and fixed with Cytofix/Cytoperm kit (BD Biosciences) and stained for IFN-γ and IL-17A following the manufacturer’s protocol. Samples were acquired on a ThermoFisher Attune NxT flow cytometer or a BD Canto flow cytometer, and data were analyzed using FlowJo (Treestar, Ashland, OR).

### ELISA

Supernatants were collected from cell culture samples at the time of cell collection. Samples were retained at − 80 °C until ELISA was performed. IL-17 ELISA (BioLegend) was performed according to manufacturer’s instructions at sample dilutions of either 2× or 5×. IFN-γ ELISA (BioLegend) was performed according to manufacturer’s instructions at sample dilutions of either 50× or 100×.

### Metabolic studies

Glucose uptake was measured using ^3^H-2-deoxy-glucose as described previously^40^. In brief, 1 million T cells were washed in Krebs–Ringer buffer (10 mM HEPES, 136 mM NaCl, 4.7 mM KCl, 1.25 mM CaCl2 and 1.25 mM MgSO4, pH 7.4). The cells were incubated for 5 min at 37 °C in the same buffer with tritiated 2-deoxy-glucose (2 μCi/reaction). The uptake reaction was stopped by adding ice-cold phloretin (Sigma-Aldrich) to a final concentration of 200 μM and centrifugation through an oil layer (1:1 Dow Corning 550 Silicon fluid (Motion Industries, Birmingham, AL) and dinonyl phthalate (Sigma-Aldrich)). The cells were lysed with 1 M NaOH, and radioactivity was measured with scintillation counting. Glucose uptake was reported as counts per minute (CPM) generated by the radioactive 2-deoxy-glucose retained in the cells. For metabolic flux assays, CD4^+^ T cells were washed with Seahorse XF RPMI 1640 media (Agilent Seahorse XF RPMI Medium with 1 mM HEPES (Agilent, Santa Clara, CA, cat#103,576–100) with 10 mM Seahorse XF Glucose (Agilent, cat#103577-100), 1 mM Seahorse XF Pyruvate (Agilent, cat#103578-100), and 2 mM Seahorse XF L-Glutamine (Agilent, cat#103579-100) and plated at a density of 250,000 cells/well (50 μL) in a Seahorse XFe96 plate (Agilent) pre-coated with Cell-Tak (Corning, Corning, NY). After spinning down the plate at 200 rpm for 1 min, the plate was incubated for 30 min in a humidified 37 °C incubator in the absence of CO_2_. 130 μL of Seahorse XF RPMI 1640 media was added, and the plate was incubated for an additional 20 min. Oxygen consumption rate (OCR) was measured using a Seahorse XFe96 Analyzer (Agilent). Mito Stress test was performed by programmed injection of oligomycin (final concentration 1 μM), FCCP (0.5 μM; dose optimized for T cells), and rotenone (0.75 μM) plus antimycin A (1 μM). To measure mitochondrial mass, 100,000 cells were plated in a V-bottom 96-well plate and stained with MitoTracker Green (MTG; 50 nM final concentration) for 30 min at 37 °C. To measure mitochondrial membrane potential, cells were stained with tetramethylrhodamine (TMRE; 100 nM) for 30 min at 37 °C. To measure mitochondrial ROS (mROS) production, cells were stained with MitoSOX Red (1.25 μM) for 30 min at 37 °C; probe specificity was confirmed using cells treated with FCCP. Samples were acquired on a ThermoFisher Attune NxT flow cytometer, and data were analyzed using FlowJo (Treestar, Ashland, OR).

### Supplementary Information

Supplementary Figures.

## Data Availability

The datasets generated during and/or analyzed during the current study are available from the corresponding author on reasonable request.
