# Supplementary material for: Insulin and IGF-1 have both overlapping and distinct effects on CD4+ T cell mitochondria, metabolism, and function
Source: Sci Rep. 2024 Feb 21;14:4331. doi: 10.1038/s41598-024-54836-w (PMC10881490; doi:10.1038/s41598-024-54836-w)
Supplement: Supplementary file 1 — Supplementary Figures. [file 41598_2024_54836_MOESM1_ESM.pdf]

Supplementary Figure S1

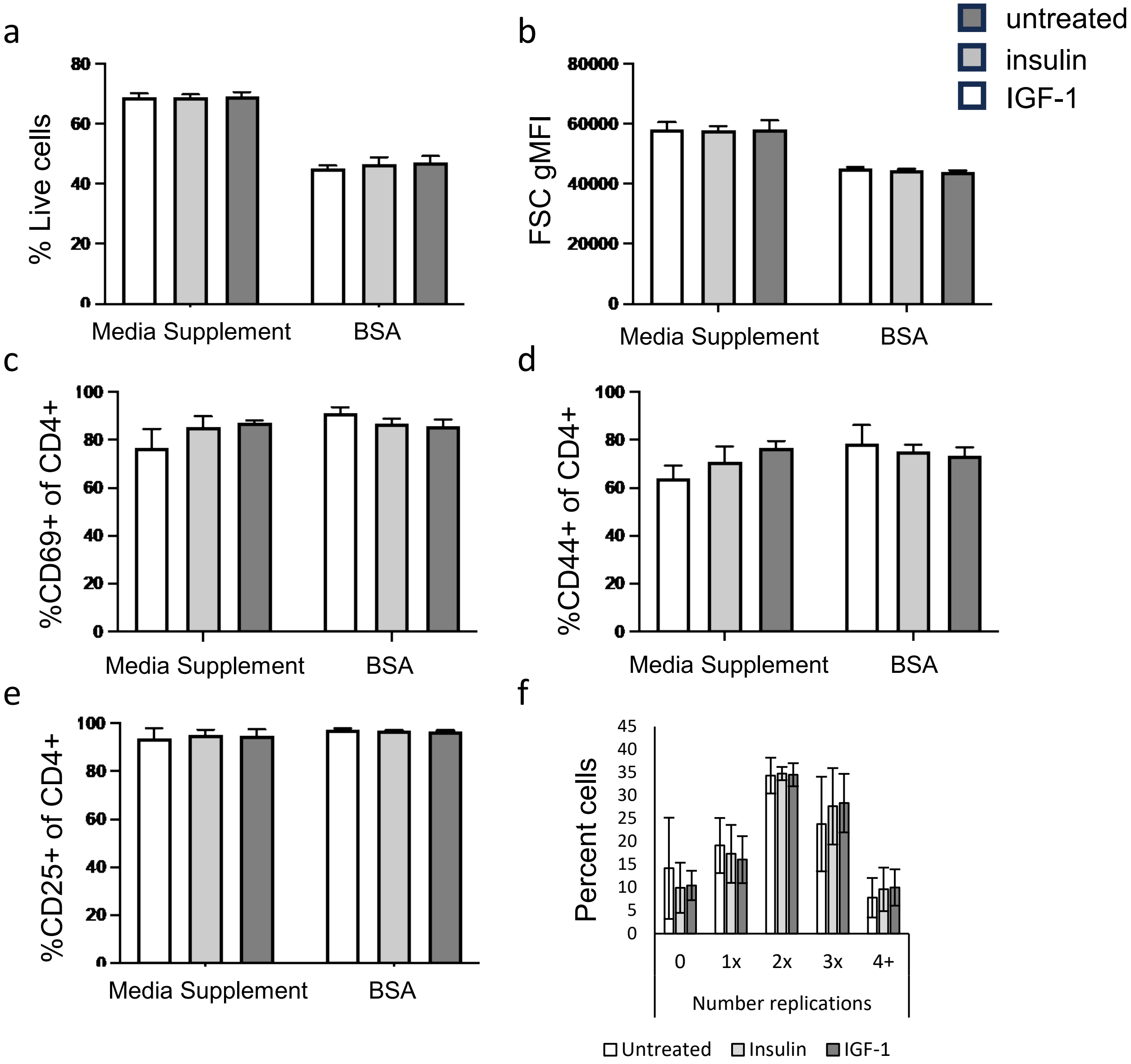

Supplementary Figure S1. Insulin and IGF-1 treatment do not impact CD4+ T cell viability, cell size, activation marker expression, or proliferation. (a-e) $CD4^{+}$ T cells were activated for 48 hours on anti-CD3/CD28 coated plates in media supplemented with either Insulin Free Media Supplement or 0.35% BSA and treated with insulin or IGF-1 for the last 24 hours of activation. Viability assessed by flow cytometry viability dye staining *(a)*. Cell size determined by FSC-A geometric mean fluorescent intensity *(b)*. Activation marker expression measured by flow cytometry for CD69 *(c)*, CD44 *(d)*, and CD25 *(e)*. **(f)** $CD4^{+}$ T cells were stained with CellTrace Violet and activated for 72 hours on anti-CD3/CD28 coated plates in serum free media supplemented with Insulin Free Media Supplement and treated with insulin or IGF-1. Proliferation assessed by flow cytometry. Data representative of at least 2 independent experiments; n=3 mice per experiment. Data analyzed using student's t-test; no significant differences found.

Supplementary Figure S2

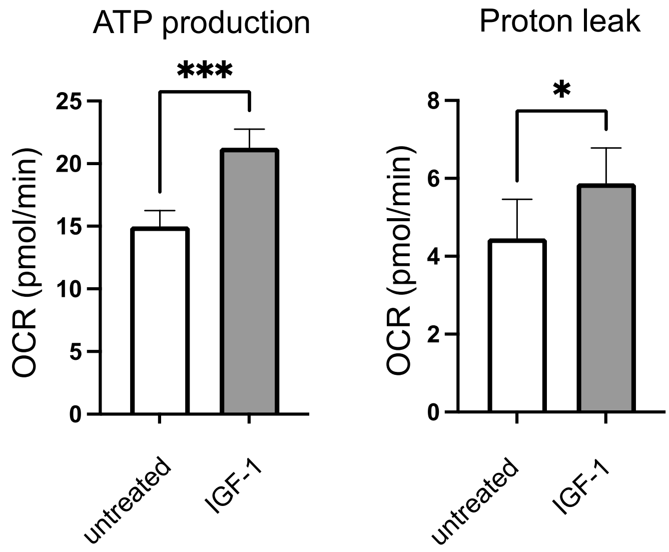

Supplementary Figure S2. IGF-1 effects on Th17 metabolism. $CD4^+$ T cells from C57BL/6 mice were differentiated *in vitro* to Th17 cells in media supplemented with Insulin Free Media Supplement for three days, and then treated with or without IGF-1 (50ng/mL) for an additional 48 hours. Extracellular flux analysis and Seahorse Mito Stress test were used to measure ATP production and protein leak. n=5-7 mice/group. Data analyzed using student's t test (*p<0.05; ***p<0.001).

Supplementary Figure S3

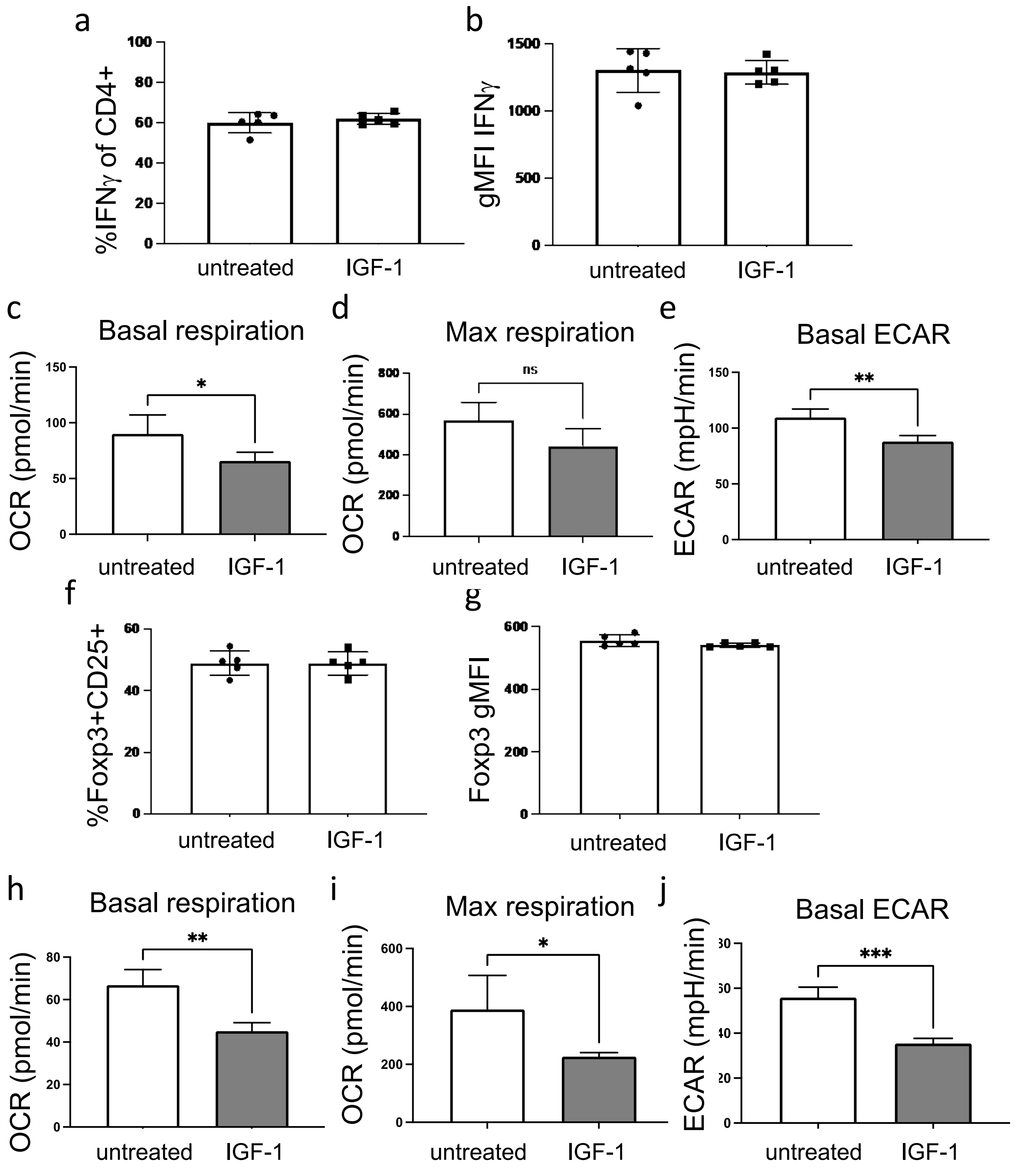

Supplementary Figure S3. IGF-1 effects on Th1 and Treg cell function and metabolism. $CD4^+$ T cells from C57BL/6 mice were differentiated *in vitro* to Th1 or Treg cells in media supplemented with Insulin Free Media Supplement for three days, and then treated with or without IGF-1 (50ng/mL) for an additional 48 hours. **(a-b)** Th1 cells were analyzed by flow cytometry for percent IFN-γ positive cells *(a)* and IFN-γ expression by MFI *(b)*. **(c-e)** Extracellular flux analysis was used to measure Th1 cell basal OCR, max OCR, and ECAR. **(f-g)** Treg cells were analyzed by flow cytometry for percent $Foxp3^+CD25^+$ positive cells *(f)* and Foxp3 expression by MFI *(g)*. **(h-j)** Extracellular flux analysis was used to measure basal OCR, max OCR, and ECAR. Data representative of 2 independent experiments. n=4-5 mice per experiment. Data analyzed using student's t test (*p<0.05; **p<0.01, ***p<0.001).

Supplementary Figure S4

Insulin receptor

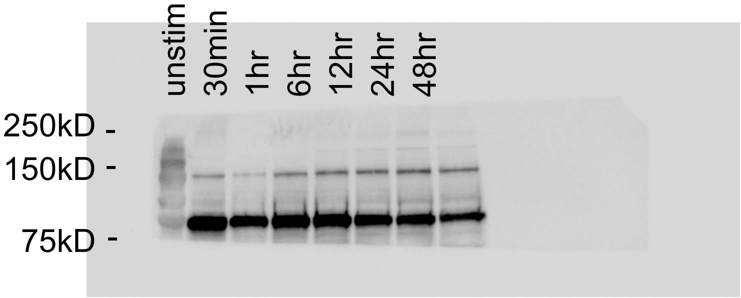

IGF1-R

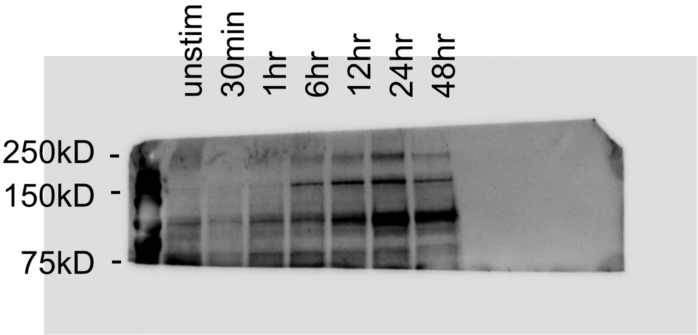

Actin

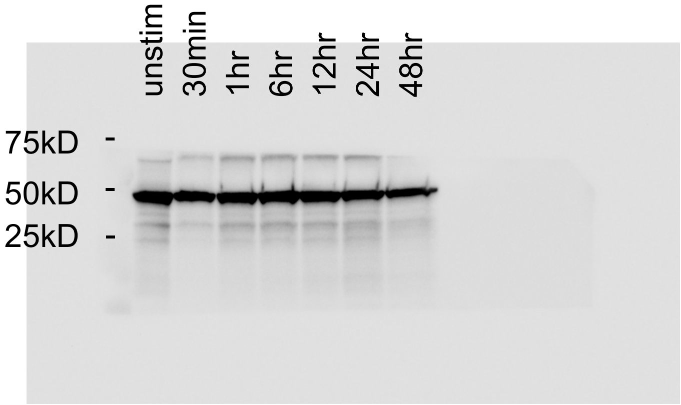

Supplementary Figure S4. Original blots used to prepare Figure 1C. IR and IGF-1R blots were stripped and reprobed; blot cut around 75kD, actin processed in parallel on the ~15-75kD strip of the blot.

Supplementary Figure S5

Insulin Receptor:

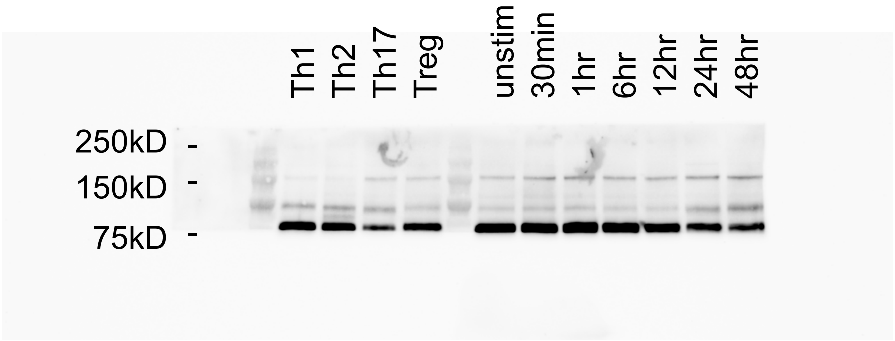

IGF-1R:

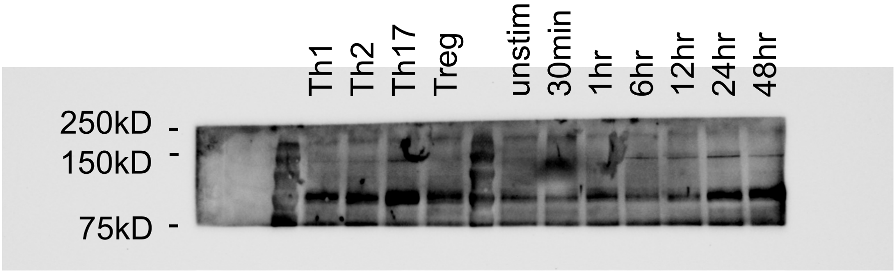

Actin:

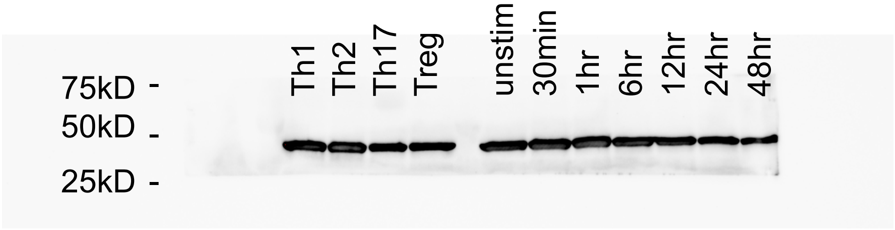

Supplementary Figure S5. Original blots used to prepare Figure 1F. IR and IGF-1R blots were stripped and reprobed; blot cut around 75kD, actin processed in parallel on the ~15-75kD strip of the blot.
